# Neurotensin as a source of cyclic AMP and co-mitogen in fibrolamellar hepatocellular carcinoma

**DOI:** 10.18632/oncotarget.27149

**Published:** 2019-08-20

**Authors:** Kimberly J. Riehle, Heidi L. Kenerson, Kevin M. Riggle, Rigney Turnham, Kevin Sullivan, Renay Bauer, John D. Scott, Raymond S. Yeung

**Affiliations:** ^1^ Department of Surgery, University of Washington, Seattle, WA, USA; ^2^ Department of Pharmacology, University of Washington, Seattle, WA, USA

**Keywords:** liver cancer, protein kinase A, fibrolamellar, neurotensin, epidermal growth factor

## Abstract

Fibrolamellar hepatocellular carcinomas (FL-HCCs) possess a unique mutation that encodes a chimeric form of protein kinase A (DNAJ-PKAc), which includes a chaperonin binding domain. DNAJ-PKAc retains most of the biochemical properties of the native enzyme, however, and activity remains dependent on cAMP. We thus speculated that a persistent source of cAMP is necessary to promote FL-HCC carcinogenesis, and that neurotensin (NTS) may drive cAMP production in this setting, given that NS serum and tumor levels are elevated in many patients with FL-HCC.

We examined expression of NTS pathway components in human FL-HCCs and paired normal livers, and determined the role of NTS in driving proliferation in tumor slice cultures. Cultured hepatocytes were used to determine interactions between NTS and other proliferative pathways, and to determine the effects of NTS on cAMP production and PKA activity.

We found that the NTS pathway is up-regulated in human FL-HCCs, and that NTS activates cAMP and PKA in hepatocytes. NTS increases proliferation in the presence of epidermal growth factor (EGF), and NTS-induced proliferation is dependent on NTSR1 and the EGFR/MEK pathway.

We conclude that NTS serves as a co-mitogen in FL-HCC, and provides a source of cAMP to facilitate ongoing activation of DNAJ-PKAc.

## INTRODUCTION

Fibrolamellar hepatocellular carcinoma (FL-HCC) is a primary liver cancer that occurs in young people without underlying liver disease. Recently, FL-HCC was mechanistically distinguished from ‘classic’ adult HCC by the discovery of the *DNAJB1-PRKACA*. This fusion transcript translates a chimeric mutant form where the DNAJ chaperonin binding domain from heat shock protein 40 is fused in frame to the catalytic subunit of protein kinase A (DNAJ-PKAc) [1, 2]. We have previously demonstrated that this mutation results in increased PKAc expression and activation in FL-HCCs, and interestingly, that mutant PKAc activity remains dependent on intracellular cAMP^3^. This finding suggests that aberrant PKAc signaling and subsequent tumorigenesis depend upon a renewable supply of upstream second messengers. One potential source of increased cAMP in FL-HCC is excess activation of upstream G-protein coupled receptors (GPCRs) and G proteins such as G_αs_, which are known to stimulate adenylyl cyclases to increase cAMP synthesis [4, 5]. G-proteins and GPCRs are key transducers of extracellular signals, and are frequently associated with human cancers [6–8].

Neurotensin (NTS) is a gut neuropeptide that is associated with a number of cancers including FL-HCC. NTS plays a role during gut development and its expression is temporally and spatially regulated [9, 10]. In the fetus, the neurotensin gene (*NT/N*) is expressed during the initial phase of intestinal and liver morphogenesis prior to cytodifferentiation. Postnatal expression of *NT/N* is restricted to the small bowel, though it is transiently expressed in the liver in the perinatal period. NTS is a tridecapeptide that has both neurotransmitter and GI endocrine functions. It is produced as a prohormone and converted into its active form by proprotein convertase subtilisin/kexin type 1 (PCSK1), a prohormone convertase [11]. Downstream effects of NTS are transduced through its engagement with GPCRs, NTSR1 and NTSR2, which in turn interact and signal through G-proteins including Gαq and Gαs. Importantly, NTS has been shown to stimulate cAMP production in certain cell types by signaling through its GPCRs, NTSR1 and NTSR2 [12, 13]. Overexpression of NTS in FL-HCC was first reported in 1984 [14]; subsequent studies have confirmed the presence of elevated serum and tumor NTS levels in these patients [15, 16]. The functional significance of this phenomenon has not been established, however, nor has a link been made between NTS and the DNAJ-PKAc fusion protein.

In this study, we examine the effects of NTS on hepatocyte proliferation *in vitro,* including in primary FL-HCC slice cultures. We confirm the overexpression of NTS, NTSR1, NTSR2, and PCSK1 in FL-HCC. We further report that NTS analogs potentiate the effects of epidermal growth factor (EGF) on hepatocyte DNA replication and cell proliferation. Importantly, this effect can be reversed by inhibition of NTSR1, EGFR, or downstream kinases. In addition, we show that NTS increases intracellular levels of cAMP as well as PKA activity in hepatocytes, to sustain DNAJ-PKAc activation in FL-HCCs. Our data suggest a role for NTS in the pathogenesis of FL-HCC, and provide a functional link between the NTS pathway and signaling by the fusion oncoprotein.

## RESULTS

### The NTS pathway is overexpressed in FL-HCC

**Figure 1:**
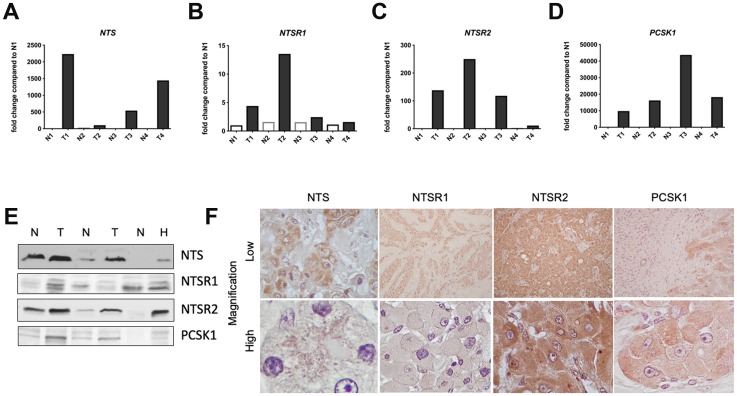
Neurotensin pathway mRNA and protein expression in FL-HCC. Relative transcript expression levels of genes expressing neurotensin (*NTS*, **A**), neurotensin receptor types 1 (*NTSR1*, **B**) and 2 (*NTSR2*, **C**), and *PCSK1* (panel **D**) in FL-HCCs (T) versus paired, normal liver (N). (**E**) Immunoblotting demonstrates expression of NTS, NTSR1, NTSR2, and PCSK1 proteins in FL-HCCs (T) vs normal livers (N). HepG2 cells (**H**) were use as positive controls. (**F**) Low (10x) and high (100x) power magnification of FL-HCCs following immunohistochemical staining for NTS, NTSR1, NTSR2, or PCSK1.

A body of clinical evidence suggests serum NTS levels are increased in patients with FL-HCC [14–18]. In light of these data, we examined the expression of components of this pathway in archived FL-HCCs from our liver tumor biorepository. qRT-PCR analysis of cDNA prepared from four FL-HCCs revealed that expression levels of *NTS, NTSR1, NTSR2,* and *PCSK1* are increased in FL-HCCs as compared to paired normal liver samples (Figure 1A–1D). Independent support for this observation was provided by immunoblotting whole tumor and adjacent liver lysates (Figure 1E, Supplementary Figure 1). Parallel analyses that investigated upregulation of each protein in tissue sections from FL-HCCs further confirmed increased expression of each protein. Furthermore, our IHC analyses suggest that it is neoplastic hepatocytes that express these NTS pathway components. IHC detection of each signaling element was low in non-parenchymal or stromal cells (Figure 1F, Supplementary Figure 2). Thus, several protein components of the NTS signaling pathway are upregulated in FL-HCC.

**Figure 2:**
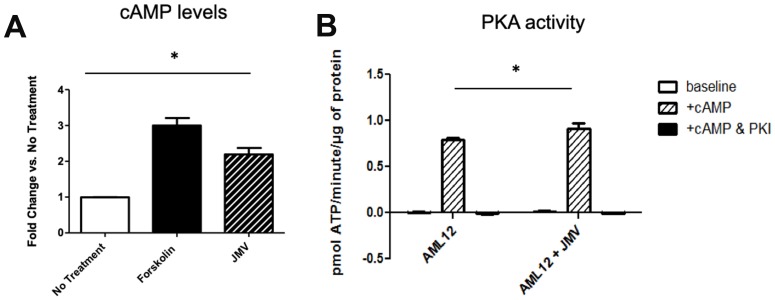
Increased cAMP release (**A**) and PKA activity (**B**) in AML 12 cells treated with neurotensin analogue JMV. Forskolin treatment is shown in (A) as a positive control; PKA activity assays were performed with and without cAMP stimulation, with demonstration of abolishment of activity with PKI, a specific inhibitor of PKA. *denotes *p* < 0.05 in cells with and without JMV treatment.

### Neurotensin activates cAMP-PKA in hepatocytes

We next hypothesized that NTS could be a source of cAMP in the liver through activation of its cognate GPCRs, NTSR1 and NTSR2. While it has been demonstrated in other systems that these GPCRs primarily signal through Gαq to activate PKC [46], their role in hepatocytes is less well defined. We made use of a non-transformed liver cell line, AML12, that retains a differentiated hepatocytic phenotype as well as the expression of the NTSRs. To determine the effects of NTS on cAMP production and PKA activity, we treated AML12 cells in culture with JMV 449 (herein referred to as JMV), a long acting NTS analog [19]. We found that JMV treatment leads to a 2.2-fold increase in cAMP levels, compared with a 3-fold increase with a potent inducer, forskolin [20], shown as a positive control (Figure 2A). To measure PKA activity, we performed radioactive *in vitro* kinase assays with Kemptide as a substrate. Analysis was conducted on fresh lysates from AML12 cells with and without JMV 449 treatment. We found that JMV treatment increases PKA activity in the presence of cAMP compared with untreated AML12 cell lysate, but only incrementally (Figure 2B). Successful blockade of kinase activity by PKI confirms that this activity is attributable to PKAc. We further examined the ability of the full-length NTS peptide (1-13) to activate cAMP and PKA in hepatocytes, and found similar trends as JMV (data not shown), thus confirming that neurotensin can induce cAMP in hepatocytes.

### Neurotensin potentiates EGF-induced cell proliferation and DNA Replication

**Figure 3:**
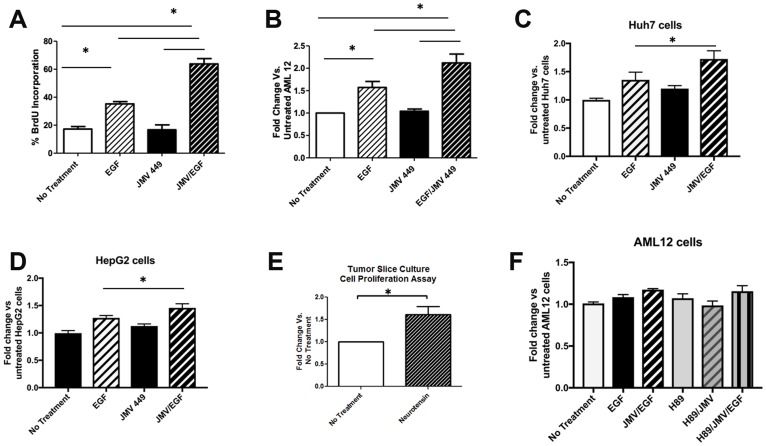
Neurotensin potentiates the effect of EGF on DNA replication and cell proliferation. (**A**) Percent of AML12 cells staining for BrdU after no treatment, EGF, the NTS analog JMV 449, or EGF + JMV. ^*^ *p* < 0.05. (**B**) AML12 cell proliferation in AML 12 cells with no treatment, EGF, JMV 449m or EGF + JMV, as measured by the MTS assay. ^*^denotes *p* < 0.05 compared to untreated AML12 cells. Proliferation of Huh7 cells (**C**) or HepG2 cells (**D**) after treatment with EGF, JMV449, or both, as measured by the MTS assay. (**E**) Neurotensin increases cell proliferation in FL-HCC slice cultures as measured by the MTS assay. F. PKA inhibition with H89 does not affect JMV/EGF-induced proliferation of AML12 cells, as measured by the MTS assay.

To determine whether increased NTS expression in liver cells has an effect on DNA synthesis and proliferation, we assessed BrdU incorporation and cell metabolic activity (based on MTT assay) in AML12 cells after NTS treatment *in vitro*. Specifically, these cells were treated with the NTS analog JMV, EGF (a known mitogen in the liver) [21, 22], or both. Interestingly, JMV alone did not stimulate DNA synthesis nor cell proliferation in AML12 cells (Figures 3A, 3B), however, it potentiated the effects of EGF, *i.e*. combination treatment induced a 3-fold increase in DNA synthesis and 1.9-fold increase in metabolic activity vs. 2-fold and 1.4-fold increases after EGF alone. To verify these findings, we tested the effects of NTS and EGF in other commonly used liver tumor-derived cell lines, Huh7 and HepG2 cells. We were able to confirm the synergistic effects of NTS and EGF in both cell lines (Figure 3C, 3D). To more directly study the effect of NTS in the intact FL-HCC microenvironment, we employed an organotypic tumor slice culture technique [23] to analyze tissue from freshly resected human FL-HCCs. Using precision-cut 250 μm x 6 mm slices grown on a solid support platform, we compared tumor slices that were placed in complete growth media containing 10 ng/mL EGF with and without JMV. We found a 1.6-fold increase in the growth rate of primary FL-HCC tumor slices after JMV treatment compared to controls (Figure 3E), further supporting the co-mitogen effect of NTS with EGF.

While JMV alone is capable of inducing cAMP and hence PKA activity (Figure 2), it fails to induce cell growth or proliferation *in vitro* (Figures 3A–3D). This suggests that PKA activity per se may not be essential in promoting liver cell growth in FL-HCC. We tested this hypothesis by repeating the above experiments using a PKA inhibitor, H89. Figure 3F illustrates the lack of change in MTT absorbance following treatment with H89 in AML12 cells treated with or without EGF/JMV. These data indicate that the synergistic effects of JMV and EGF are not mediated through PKA kinase activity.

### Pathways activated by Neurotensin/EGF in hepatocytes

**Figure 4:**
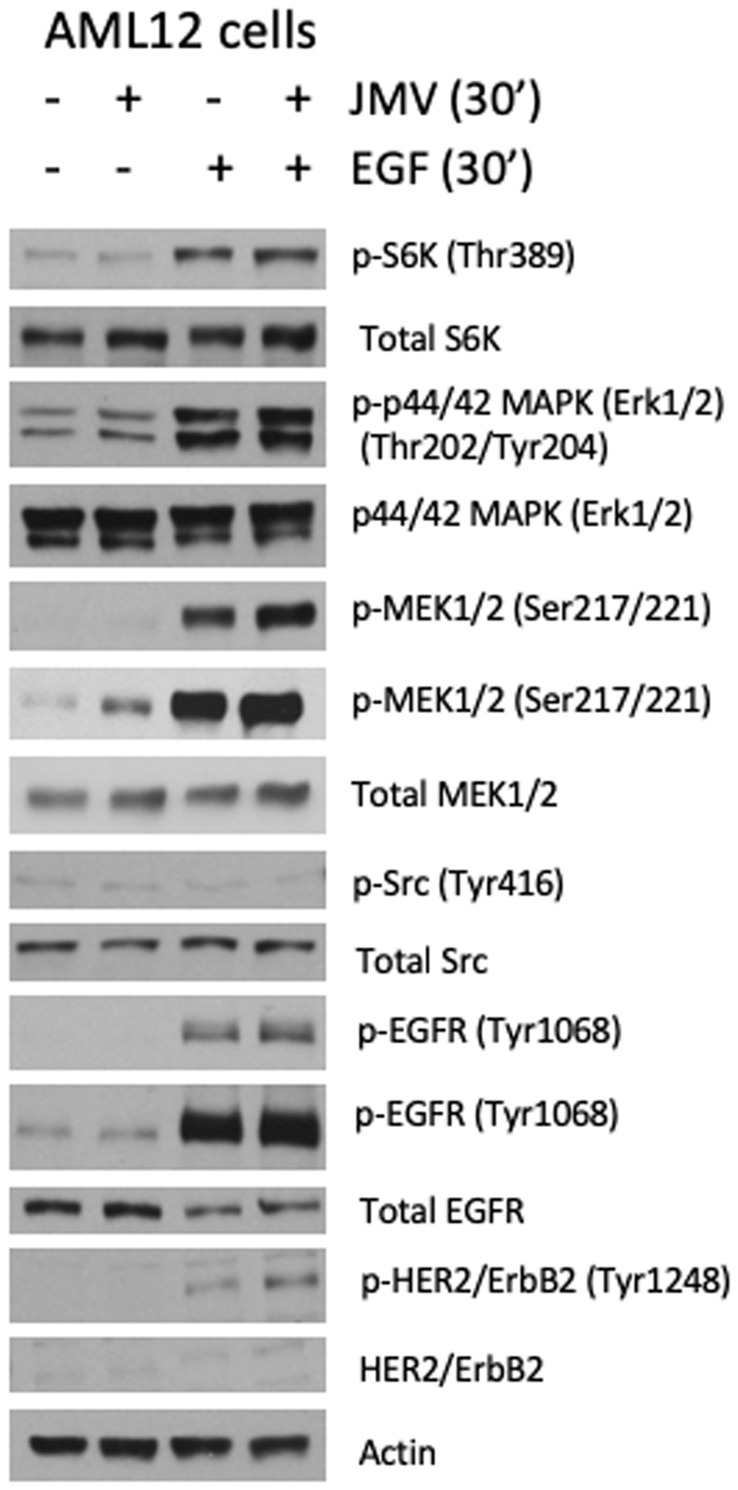
JMV/EGF activate ERK 1/2 and MEK. Immunoblotting of AML12 cell lysates with or without treatment with EGF, the NTS analog JMV 449, or both, as indicated at the top of the figure, using primary antibodies for phospho- and total S6 kinase, phospho- and total p44/42 MAPK (ERK 1/2), phospho- and total MEK 1/2, phospho- and total Src, phospho- and total EGFR, and phospho- and total HER2/ErbB2. Actin is shown as a loading control.

To define the signaling events downstream of NTS and EGF in hepatocytes, we performed immunoblot analyses on AML12 cell lysates following exposure to either or both ligands. Figure 4 demonstrates activation of molecular pathways known to promote cell growth and proliferation the liver after treatment with JMV, EGF or both, including ribosomal S6 kinase (S6K), mitogen activated protein kinase (MAPK/ERK), mitogen-activated protein kinase kinase (MEK) and Src. As expected, stimulation of hepatocytes with EGF engages its receptors, EGFR and HER2 resulting in autophosphorylation, which in turn activates downstream mitogenic pathways, including S6K and MAPK. Treatment of hepatocytes with JMV alone induces only a modest elevation in MEK phosphorylation, without evidence of S6K or Src activation. However, when used in combination with EGF, JMV further increases EGF receptor (EGFR), HER2 and MEK phosphorylation, without affecting S6K and Src phosphorylation. These findings suggest that NTS potentiates MAPK signaling induced by EGF, serving as a co-mitogen in hepatocyte proliferation.

### Neurotensin/EGF-induced proliferation is abrogated by inhibition of NTSR1, EGFR, or MEK

**Figure 5:**
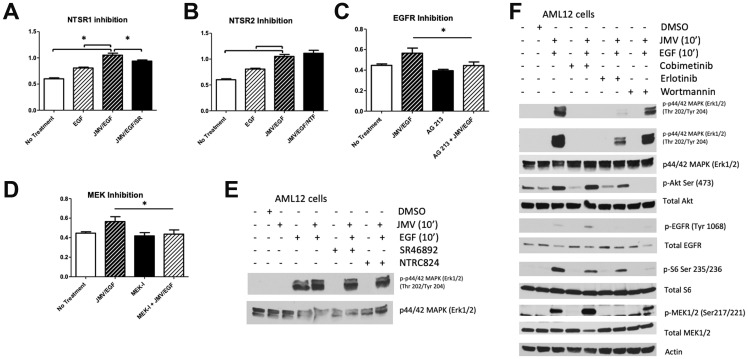
Effect of inhibitors on JMV/EGF-induced proliferation in AML12 cells. JMV/EGF-stimulated AML12 cell proliferation was blocked by pretreatment with an inhibitor to NTSR1 (SF46892, **A**), but not NTSR2 (NTFC824, **B**). Either EGFR (AG213, **C**) or MEK (cobemetinib, **D**) inhibition block the proliferative effects of JMV/EGF treatment in AML12 cells. (**E**) Neither pre-treatment with NTSR1 (SR46892) or NTSR2 (NTFC824) inhibitors change ERK1/2 phosphorylation in response to EGF/JMV treatment. (**F**) Inhibition of EGF (erlotinib) or MEK (cobimetinib) decreases ERK1/2 phosphorylation in response to JMV and EGF; PI3K inhibition (wortmannin) does not affect ERK1/2 activation. Activation levels of Akt, EGFR, total S6, and MEK are also shown, with Actin serving as a loading control.

To determine which of the two NTS receptors expressed in AML12 cells is responsible for the synergistic effects of NTS on EGF, we treated AML12 cells with a small molecule inhibitor for NTSR1 (SR 46892) or NTSR2 (NTRC 824) for 30 minutes prior to the addition of JMV and/or EGF. Pharmacological inhibition of NTSR1 blunted the JMV/EGF-induced proliferative effect, while inhibition of NTSR2 had no effect (Figure 5A, 5B). To further delineate the role of EGFR and its downstream signaling pathways in JMV/EGF-induced cell proliferation, we treated AML 12 cells with PP2 (Src inhibitor), erlotinib (EGFR inhibitor), cobimetinib (MEK inhibitor), Calphostin C (PKC inhibitor), wortmannin (PI3K inhibitor), or DMSO control 30 minutes prior to the addition of JMV and/or EGF. We found that inhibiting either EGFR or MEK blocked the proliferative effects of JMV/EGF (Figure 5C, 5D). By contrast, inhibition of SRC, PKC, or PI3K had no effect on JMV/EGF induced proliferation (data not shown). Figure 5E demonstrates that inhibition of NTSR1 or NTSR2 does not affect ERK activation significantly. Conversely, erlotinib and cobimetinib blocked activation of ERK, while wortmannin treatment has no effect on ERK phosphorylation, as expected (Figure 5F). Taken together, our results demonstrate that NTS has a co-mitogen effect on EGF signaling in cultured hepatocytes by enhancing EGFR phosphorylation and activation of MAPK signaling pathways. These data allow us to propose a plausible mechanism whereby NTS supports tumor growth in FL-HCC, in addition to its ability to stimulate cAMP release and thus PKA activity.

## DISCUSSION

The recent discovery of the *DNAJB1-PRKACA* fusion transcript in FL-HCC has been a major advancement in our understanding of the pathogenesis of this deadly cancer [1–3, 5, 24–26]. We have previously described overexpression of the resultant fusion protein, DNAJ-PKAc, in these tumors, and that PKAc kinase activity remains dependent on cAMP [3]. Specifically, we found that basal PKA activities of FL-HCCs are similar to that of adjacent non-tumor livers, but in the presence of the same amount of cAMP, tumor PKA activities are significantly higher than that of normal liver due to elevated expression of DNAJ-PKAc in FL-HCCs. We thus concluded that there must be a basal supply of cAMP in order to drive PKAc activity in FL-HCCs [2]. In creating a mouse model of this disease, Kastenhuber *et al* highlight the importance of PKA activity in tumor development since the over-expression of a ‘kinase-dead’ DNAJ-PKAc failed to induce liver tumors [27]. In this study, we show that NTS and its GPCR, NTSR1, are a potential source of cAMP in FL-HCC. Our data indicate that NTS elevates cAMP and hence PKA activity in hepatocytes consistent with previous findings in other, non-hepatocyte, cell lines [12, 13, 28]. In keeping with our hypothesis, FL-HCCs have up-regulated expression of NTS, its prohormone convertase, PCSK1, and its receptors, NTSR1 and NTSR2. These findings are consistent with the known phenomena of hyper-neurotensinemia in patients with fibrolamellar cancer [15, 17], and provide a novel link between NTS and the DNAJ-PKAc fusion protein.

Although we do not yet know the precise molecular mechanism through which the NTS cascade is affected in FL-HCC, we propose that co-expression of the ligand, its receptors and processing protein in the same tumor cells (Figure 1F) is key to this pathological process. Moreover, these findings raise the possibility of an autocrine/paracrine loop in which NTS, through the engagement with its GPCRs, promotes the transcription of the *NTS* gene, leading to increased production, processing and packaging of mature peptide hormones bound for vesicle-mediated secretion. Studies have shown that PKA induces NTS secretion, and that NTS expression is positively regulated by cis-elements in its promoter that bind CREB, a putative target of PKA [29]. Additionally, transcriptomic analyses of FL-HCC highlight a neuroendocrine signature [30] that may be reflected in an increased synthesis and turnover of the neurotensin-containing secretory granules (Figure 1F). It is important to recognize that other peptide hormones may synergize with NTS to play a role in FL-HCC, yet there has been no systematic analyses of candidates that may be relevant to liver pathophysiology to date. We are aware that the intracellular effects of PKA signaling are highly pleiotropic, and determined by cell type, sub-cellular localization of the holoenzyme, and proximity to substrates [31, 32]. In many contexts PKA signaling inhibits proliferation and promotes differentiated cellular functions [33, 34]. More work must be done to determine the precise mechanism through which DNAJ-PKAc signaling drives tumor formation in otherwise normal livers.

In addition to the effects of NTS on cAMP signaling, we show that NTS potentiates the effects of EGF on hepatocyte proliferation. We provide evidence that NTS alone is not mitogenic to hepatocytes in culture, and treatment with a PKA inhibitor, H89, has no influence on cell growth. These findings infer that NTS may act as a co-mitogen independent of PKA activity. Depending on the cell type, studies have highlighted the role of PLC and PKC downstream of GPCRs in the trans-activation of EGFRs [35], via signaling through Gαq. Other investigators indicate that the generation of heparin-binding EGF (HB-EGF) is localized to the plasma membrane through the action of membrane type-1 MMP that is unaffected by inhibitors of PLC and PKC [36]. Regardless of the precise mechanism involved, there is general agreement that NTS induces an autocrine/paracrine stimulation of EGFRs through cleavage and shedding of EGF-like ligands (*e.g*. HB-EGF) [37]. Multiple groups have demonstrated upregulation of the EGF pathway in FL-HCCs showing increased expression of EGF as well as its receptors EGFR and ErbB2 [17, 26, 38]. That NTS serves as a co-mitogen of EGF has also been observed in multiple cell types [21, 39]. Based on our protein kinase inhibitor data, the EGFR pathway seems paramount for this EGF/NTS-induced cell proliferation, at least in cultured hepatocytes. This effect on the EGFR may be a combination of Gαs signaling through PKA and Gαq signaling through PKC, as either mechanism may result in EGFR transactivation [46]. We found that inhibition of the EGF pathway with either erlotinib (EGFR inhibitor) or cobimetinib (MEK inhibitor) abrogated EGF/NTS induced cell proliferation. Other pathways including SRC and PKC have been implicated in NTS-mediated proliferative effects in both colon and pancreatic cancers [40–42]; we failed to show any inhibition of the proliferative effects of EGF/NTS through inhibition of these kinases. Interestingly, in animal and cell models for NTS-expressing lung cancers, treatment with an NTSR1 antagonists resulted in increased cytotoxicity of anti-EGFR chemotherapy [22]. Our findings in AML12 hepatocytes noted that the effects of NTS were partially inhibited by NTSR1, but not NTSR2, antagonists. These data suggest that while EGFR inhibitors have thus far failed to show any benefit in treating FL-HCCs [43], perhaps combined inhibition of NTS and EGFR or MEK inhibition would provide a therapeutic benefit to these patients.

In summary, this report highlights the biologic significance of neurotensin in FL-HCC by serving two important functions: 1) NTS provides a source of cAMP to stimulate PKA activity in the initiated tumor cells, and 2) acting through NTSR1, NTS potentiates cell growth and proliferation through the EGFR pathway. Further study into the precise interactions between NTS, EGF, and DNAJ-PKAc must be undertaken to fully understand the pathogenesis of FL-HCC. Unfortunately, adequate animal models of FL-HCC are currently lacking and studying the precise effects of the *DNAJB1-PRKACA* fusion transcript in non-transformed hepatocytes has proven difficult. Nonetheless, we anticipate that with the generation of FL-HCC specific models and mapping out the effector pathways activated by NTS and DNAJ-PKAc, targeted therapies for patients with FL-HCC will become a reality.

## MATERIALS AND METHODS

### Human liver samples

Human FL-HCCs and paired normal livers were obtained from the University of Washington Medical Center and Seattle Children’s Hospital after institutional review board approval (SCH IRB #15277). For prospective fresh tissue collections, informed consent was obtained from the subject and/or parent prior to resection.

### RNA extraction and RT-qPCR

RNA was extracted from tumors and surrounding liver using TRIzol (Life Technologies, Grand Island, NY) as described by the manufacturer. qRT-PCR was performed with the following FAM labeled primers: *NTS*, *NTSR1*, *NTSR2*, *PCKS1*, *GNAS, GNAQ, and 18S* (Life Technologies). qRT-PCR reactions contained cDNA synthesized from 1.0 μg RNA using MMLV reverse transcriptase (Life Technologies), and TaqMan (Life Technologies) master mix. Cycling conditions were 95°C for 10 min, and 39 cycles of 95°C for 15 sec and 60°C for 60 sec, with a final extension at 72°C for 1 min in an Opticon 2 thermocycler (MJ Research Incorporated, St. Bruno, Quebec, Canada). Data are represented as delta delta Ct values after normalization to *18S* mRNA levels.

### Immunoblotting

Human FL-HCCs, normal liver samples, and hepatocyte cultures were homogenized in ice-cold radioimmunoprecipitation (RIPA) buffer containing protease inhibitors, and protein concentrations were measured using the BCA Protein Assay (Pierce, Rockford, IL). Equal amounts of protein were separated by SDS-PAGE, transferred to Immobilon-P membranes (Millipore, Bedford, MA) and incubated at 4 degrees C overnight with the following primary antibodies: NTS (sc-20806, Santa Cruz), NTSR1 (sc-376958, Santa Cruz), NTSR2 (NB100-56472, Novus Biologicals), PCSK1 (sc-100578, Santa Cruz), phospho-p70 S6 Kinase (Thr389, #97596), phospho-p42/44 (#4370), phospho-MEK1/2 (Ser217/221, #9154), phospho-Src (Tyr416, #2101), phospho-EGFR (Tyr1068, #3777), phospho-HER2 (Tyr1248, #2247; all Cell Signaling) and β-Actin (A5441, Sigma, St. Louis, MO).

### Immunohistochemistry (IHC)

IHC analyses were conducted on formalin-fixed, paraffin-embedded sections of human FL-HCC. After deparaffinization, rehydration, and quenching, antigen unmasking was conducted in 0.01 M citrate buffer of pH 6.0 in a microwave for 10 min. After incubation with the primary antibodies listed above, sections were developed with an avidin–biotin technique using the VECTASTAIN Elite ABC kit (Vector Laboratories, Burlingame, CA) and counterstained with Gill’s hematoxylin.

### Cell culture experiments

Non-transformed mouse hepatocytes (AML12 cells) [44] were grown in Dulbecco’s modified Eagle Medium (DMEM) F12 with insulin (5 ug/mL), transferrin (5 ug/mL), selenium (5 ug/mL), dexamethasone (0.04 ug/mL), 0.1% gentamicin, and 10% fetal bovine serum (FBS). Confluent cells were plated in a 24-well plate at a concentration of 4.0 x 10^4^ cells per well and serum-starved the next day. Huh7 and HepG2 cell lines were similarly cultured per standard protocols. Following 12 hours of serum starvation, cells were treated with 10 ng/mL EGF, 1 μM JMV449, or EGF and JMV together, and incubated for 48 hours. For receptor and kinase inhibitor experiments, cells were pre-treated with SR46892 (NTSR1 inhibitor), NTRC824 (NTSR2 inhibitor), PP2 (Src inhibitor), AG213 (EGFR inhibitor), erlotinib (EGFR inhibitor), cobimetinib (MEK inhibitor), wortmannin (PI3K inhibitor) calphostin C (PKC inhibitor), or DMSO control 30 minutes prior to addition of JMV449 and/or EGF.

Cell proliferation was measured by the 3-(4,5-Dimethylthiazol-2-yl)-2-5-diphenyltetrazolium bromide (MTT) assay. 5 mg/mL MTT in PBS was added to each well; cells were incubated at 37°C, 5% CO_2_ for 3 hours. Media were removed, and MTT solvent (4 mM HCl, 0.1% Nonidet P-40 in isopropanol) was added to each well to dissolve the MTT formazan crystals. Cells were incubated at room temperature in the dark on a rocker for 15 minutes. Absorbance of triplicates from each sample was measured at 590 nm.

### BrdU immunocytochemistry

To measure DNA replication, AML12 cells treated as described above were incubated with 3 ng/mL BrdU. After incubation with BrdU for 10 hours, cells were fixed in 90% ethanol, 5% 17.4 N acetic acid, 5% dH2O for 30 minutes at room temperature. Following fixation, cells were quenched and antigen unmasking performed in 1.5 N HCl for 15 minutes at 37° C. After incubation with anti-BrdU primary antibody, cells were developed using an avidin–biotin technique using the VECTASTAIN Elite Mouse IgG kit (Vector Laboratories; Burlingame, CA), and counterstained with Gill’s hematoxylin.

### Tumor slice culture and proliferation assay

To more directly study the effect of NTS in primary FL-HCCs, we employed a primary tumor slice culture technique to directly assay freshly resected FL-HCCs (*n =* 2). Following resection, intact tumor tissue was precisely cut using a Leica VT 1200 S Vibrating Microtome (Leica; Germany) to obtain slices measuring 250 μm thick and 6 mm in diameter. Tissue slices were cultured on transwell plates (Millipore; Darmstadt, Germany) with growth media consisting of William’s E medium supplemented with nicotin-amide (10 mmol/L); HEPES (20 mmol/L); NaHCO_3_(17 mmol/L); pyruvate (550 mg/L); ascorbic acid-2-phos-phate (0.2 mmol/L; Sigma); glucose (14 mmol/L); glutamine (2 mmol/L); 10^^^7mol/L dexamethasone (Sigma); ITS pre-mix containing insulin (6.25 μg/mL), transferrin (6.25 μg/mL), selenious acid (6.25 ng/mL), bovine serum albumin (1.25 mg/mL), and linoleic acid (5.35μg/mL;Becton-Dickinson, Bedford, MA); and 5% fetal bovine serum (Hyclone Corp; Logan, UT). After 2 days in culture, tumor slices were treated with NTS and cell proliferation was measured using the Cell Titer 96^®^ AQueous One Solution Cell Proliferation Assay (MTS) according to the manufacturer’s protocol (G3581, Promega; Madison, WI).

### cAMP assay

In order to measure the effects of NTS on hepatocyte cAMP production, we cultured AML12 cells as described above in 6-well plates. After cells were 70% confluent, they were treated with 1 uM JMV 449, 1 uM NTS (1–13), 2 uL DMSO, or no treatment for 30 minutes. 10 uM forskolin was used as a positive control for cAMP production. At the end of 30 minutes we lysed the cells in lysis buffer from the cAMP ELISA kit (STA-500, Cell Biolabs; San Diego, CA), and performed the assay according to the manufacturer’s protocol.

### Kinase assays

Cell lysates were prepared after stimulation with JMV as described above, and subjected to radioactive kinase activity assays, which were performed in triplicate with Kemptide as a substrate. Assays were performed in with or without 5 μmol/l cAMP and/or PKI, a specific inhibitor of PKA as described [45].

### Statistical analyses

Statistical analysis was performed using GraphPad Prism (GraphPad Software) on all data, using the paired Student *t*-test and analysis of variance with post hoc test where appropriate, with a *p* value ≤ 0.05 considered to be significant.

## SUPPLEMENTARY MATERIALS



## Abbreviations

FL-HCC: fibrolamellar hepatocellular carcinoma
PKAc: catalytic subunit of protein kinase A
NTS: neurotensin
EGF: epidermal growth factor
GPCRs: G-protein coupled receptors
PCSK1: proprotein convertase subtilisin/kexin type 1
JMV 449: H-Lys psi (CH2NHLs-Pro-Tyr-Ile-Leu-OH
S6K: ribosomal protein S6 kinase
MAPK: mitogen activated protein kinase
ERK: extracellular signal-regulated kinase
PKC: protein kinase C
PI3K: phosphoinositide 3-kinase
HB-EGF: heparin-binding epidermal growth factor-like growth factor
DMEM: Dulbecco’s modified eagle medium
MTT: 3-(4,5-dimethylthiazol-2-yl)-2,5-diphenyltetrazolium bromide
