# Supplementary material for: Neurotensin as a source of cyclic AMP and co-mitogen in fibrolamellar hepatocellular carcinoma
Source: Oncotarget. 2019 Aug 20;10(49):5092–102. doi: 10.18632/oncotarget.27149 (PMC6707953; doi:10.18632/oncotarget.27149)
Supplement: Supplementary file 1 [file oncotarget-10-5092-s001.pdf]

Neurotensin as a source of cyclic AMP and co-mitogen in fibrolamellar hepatocellular carcinoma

SUPPLEMENTARY MATERIALS

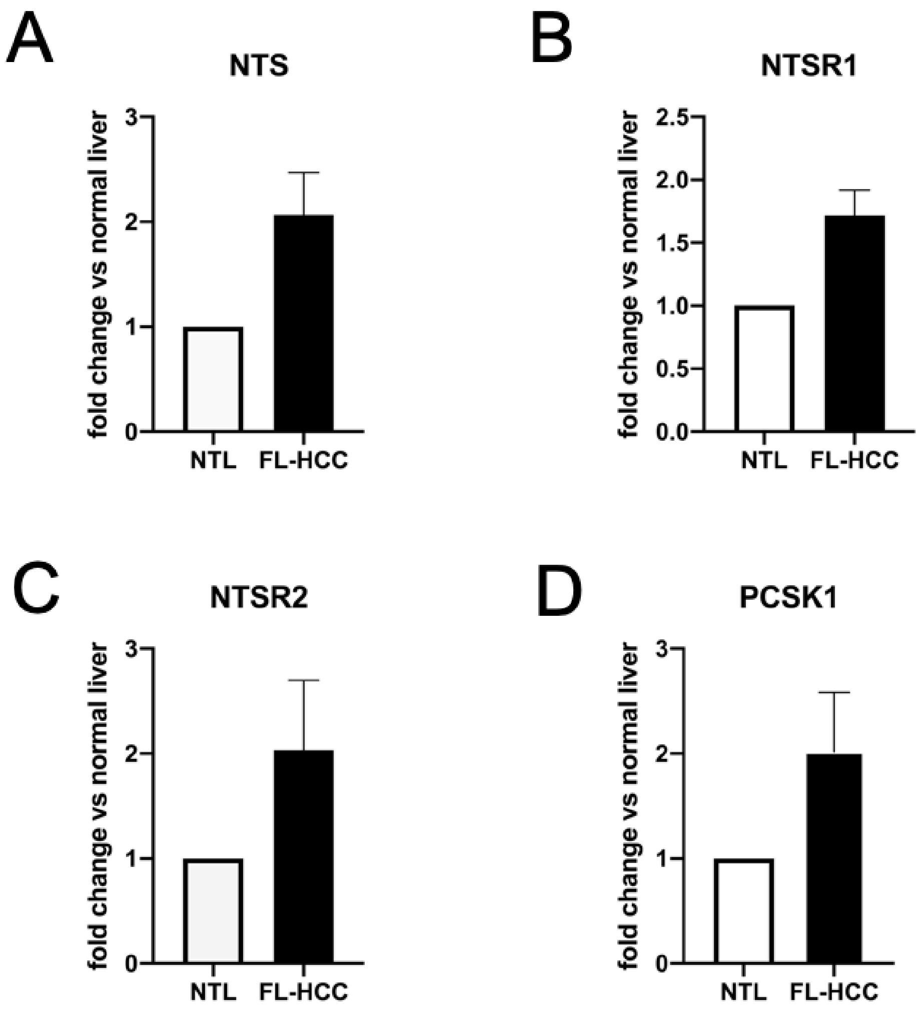

Supplementary Figure 1: Overexpression of NTS pathway components in FL-HCC. Immunoblotting was performed on FLCs and paired non-tumor livers (*n* = 3–4) and densitometric analysis was performed, confirming increased protein expression of NTS (**A**), NTSR1 (**B**), NTSR2 (**C**), and PCSK1 (**D**).

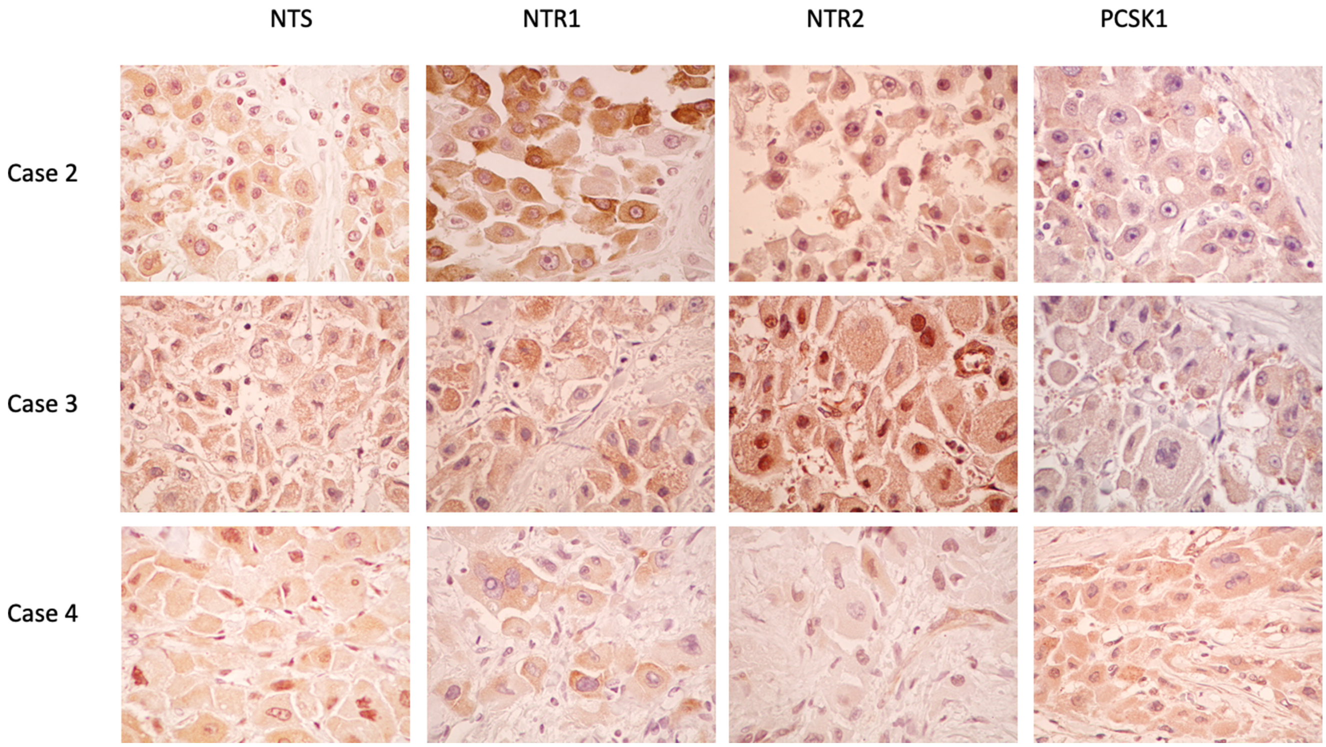

Supplementary Figure 2: Additional immunohistochemical analyses of FL-HCC sections.
